# Exploring the interplay between dietary plant glycolipids and gut microbiota: A systematic review

**DOI:** 10.1080/29933935.2026.2676398

**Published:** 2026-09-26

**Authors:** Anastasia Cartegnie, Eva Larrieu, Marie-Caroline Michalski, Cécile Vors

**Affiliations:** a Université Lyon 1, INSERM, INRAE, CarMeN, Pierre-Bénite, France; b Lyon GEM Microbiota Study Group, Lyon, France

**Keywords:** Green foods, galactolipids, gut bacteria, prebiotics, plant-based diets

## Abstract

Considering the shift towards plant-based diets, it becomes necessary to better understand the roles of plant-derived nutrients in health. Despite their abundance in green plants, the role of plant glycolipids (GLs) in metabolic health remains scarcely known. Gut microbiota is a key actor in the regulation of host health, whose interplay with diet is complex. This systematic review examines the available evidence on potential interactions between dietary plant GLs and the gut microbiota. A structured search strategy was used to identify preclinical and human studies assessing the impact of plant GLs on the gut microbiota composition or the potential degradation of plant GLs by gut bacteria. Among 345 screened records, 13 studies were finally included. Both animal- and human-based evidence suggest that plant GLs may impact the gut microbiota composition, with beneficial effects for host by promoting a healthy gut bacterial ecosystem (increase in gut health-related bacteria and a reduction in some pathobionts). Some GLs exhibit *in vitro* antibacterial properties, and several pathways of GL bacterial degradation have been described, suggesting that the gut microbiota uses plant GLs as a source of nutrients. Such promising evidence highlights the potential prebiotic role of plant GLs and encourages further study of the gut-associated metabolic effects of plant GLs in humans.

## Introduction

The current scientific consensus is to move towards a “sustainable healthy diet” (as defined by FAO, 2019) to combine health and environmental issues. The EAT–Lancet Commission recommends a daily intake of approximately 500 g of fruits and vegetables as part of a healthy and sustainable diet,^1^ a recommendation that has been reaffirmed and further supported in the most recent EAT–Lancet Commission report.^2^ Diets based largely on plant-based foods are most associated with health benefits and longevity.^3^ To explain these favorable associations, many studies have assessed the direct impact of plant proteins and different phytonutrients (e.g. polyphenols, flavonoids, carotenoids) on human cardiometabolic health.^4^ ^,^ ^5^ In parallel, research on the gut microbiota has increasingly grown in order to better understand the diet-host-gut microbiota interactions and their key role in metabolic health.^6^ ^,^ ^7^ In this respect, dietary fibers are the most studied plant-based food components so far because they are fermentation substrates for gut bacteria, generating short chain fatty acids which play crucial roles for host intestinal and metabolic health.^8^ Other bioactive plant-based molecules could also be considered as potential prebiotics,^9^ in particular regarding some specific lipids of plants whose health effects are poorly explored to date, namely glycolipids (GLs).

Plant GLs are found in the photosynthetic plant kingdom, representing 70% to 80% of total lipids of plant cells.^10^ They are present in grains, roots, and fruits but they are particularly concentrated in the thylakoid membranes of chloroplasts within plant leaves. Green fruits and vegetables are therefore important edible sources of such lipids.^11^ GLs mainly consist of monogalactosyldiacylglycerol (MGDG), digalactosyldiacylglycerol (DGDG) and sulfoquinovosyldiacylglycerol (SQDG), which constitute about 50%, 25%, and 7% of the total lipids in thylakoids, respectively.^12^ Plant GLs are classified as polar lipids owing to their specific molecular structure, which is composed of a hydrophilic (polar) sugar headgroup and a hydrophobic (nonpolar) lipid moiety (Figure 1). Plant GLs are characterized by two saturated and/or unsaturated fatty acids with chain lengths typically varying from C16 to C20 linked to the glycerol moiety. Their composition also varies according to the nature and number of sugar residues in the polar head, i.e. MGDG and DGDG bearing one or two galactoses, respectively, and SQDG bearing a sulfoquinovose (SQ) (Figure 1). Of note, MGDG and DGDG are called “galactolipids” because of their galactose component. Plant GLs in the thylakoid membranes of green vegetables are characterized by their high content of polyunsaturated fatty acids (PUFAs),^13^ especially the GLs that are naturally rich in omega-3 α-linolenic acid (ALA; 18:3 *n* − 3) (Figure 1), a long-chain essential PUFA.

**Figure 1.**
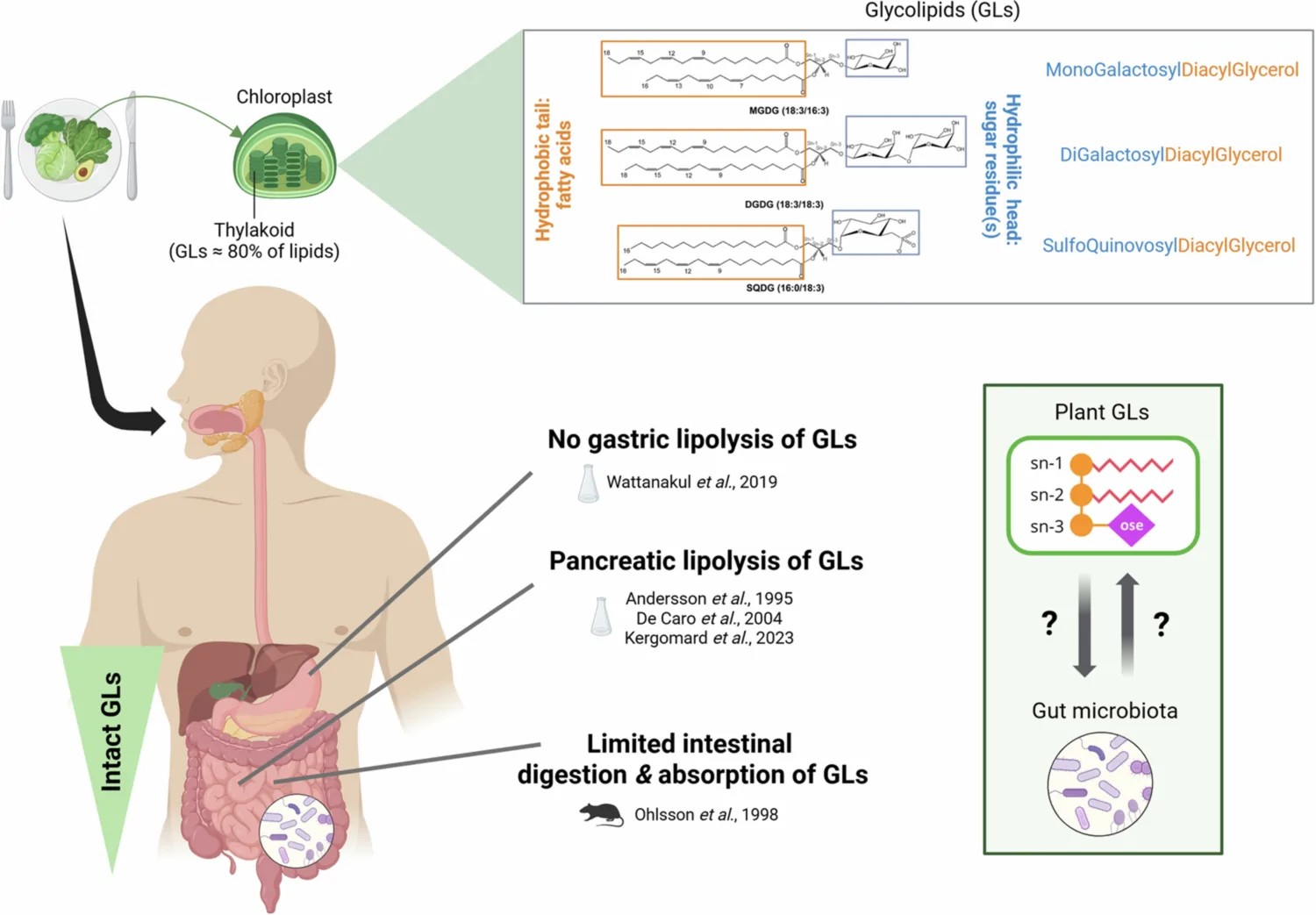
Schematic representation of plant glycolipid (GL) sources and molecular structures, as well as their fate along the gastro-intestinal tract, suggesting potential interactions with the gut microbiota. GLs are concentrated in the chloroplast fold membranes, named thylakoids, of green plant-based foods. GLs are polar lipids with a hydrophilic sugar headgroup and a hydrophobic lipid moiety (fatty acids). Molecules of monogalactosyldiacylglycerol (MGDG), digalactosyldiacylglycerol (DGDG) and sulfoquinovosyldiacylglycerol (SQDG) are represented with predominant fatty acid composition in spinach leaves. GLs do not undergo gastric lipolysis,^14^ but two pancreatic enzymes, pancreatic lipase-related protein 2 (PLRP2) and carboxyl ester lipase (CEL), have been described as having specific lipolytic activity on GLs.^15-17^ Rat studies using radiolabeled GLs showed however a limited intestinal digestion and absorption of GLs.^18^ Intact GLs could thus reach the distal part of the intestine and interact with the gut microbiota. Created with BioRender.com.

Recent research has shown that residual dietary lipids reaching the distal intestinal part can modulate the gut microbiota composition and/or functionality.^19-21^ Regarding plant GLs, they were shown to not undergo gastric lipolysis^14^ (Figure 1). A lipolytic activity of human pancreatic enzymes (pancreatic lipase-related protein 2, PLRP2; carboxyl ester lipase, CEL) on GLs has been shown *in vitro.* ^15-17^ However, *in vivo,* it appears that GL may not be completely digested and absorbed, as rat studies using radiolabeled DGDG have shown that (1) labeled fatty acids from GLs were only partially absorbed (<15% recovery in chyle) and (2) intact labeled-GLs were found to be present in the distal part of the rat intestinal tract^18^ (Figure 1). As such, intact GLs would thus be able to interact with the gut microbiota. Additionally, owing to their particular structure, plant GLs are natural vectors of omega-3 α-linolenic acid, which can be metabolized by gut lactic acid bacteria into anti-inflammatory compounds,^22^ and of specific carbohydrates, such as sulfoquinovose that can be preferentially consumed by gut commensal bacteria as an energy source.^23^ Therefore, the present systematic review aimed to summarize the available evidence allowing to answer the following questions: do plant GLs have specific interactions with the gut microbiota? May plant GLs have an impact on the intestinal microbiota composition? Are gut bacteria able to metabolize plant GLs? To examine the relationships between plant GLs and the gut microbiota, we focused on (1) the potential impact of dietary plant GLs on the composition of the gut microbiota and (2) the degradation of plant GLs by gut bacteria.

## Methods

The present systematic review was conducted according to the Cochrane Handbook for Systematic Reviews of Interventions.^24^ The results are reported in accordance with the Preferred Reporting Items for Systematic Reviews and Meta-Analyses (PRISMA).^25^

### Search strategy

The literature search was conducted through the “Web of Science” database until March 20th 2023. In line with PRISMA guidelines, Web of Science was used to ensure comprehensive coverage of the literature across disciplines, allowing the identification of relevant clinical, preclinical, and mechanistic studies that may be underrepresented in more restrictive, clinically oriented databases (e.g. MEDLINE/Pubmed, Cochrane). The literature search was divided into two parts: the first search (search 1) was based on keywords related to the potential impact of GLs or foods rich in GLs on the gut microbiota composition, and the second (search 2) focused on literature studying the degradation of GLs by gut bacteria (excluding literature on foods rich in GLs). Therefore, the list of keywords in search 1 focused mainly on search terms related to plant GL names/structures and GL food sources and the gut microbiota, whereas the search terms in search 2 focused on GL names/structures and the gut microbiota/bacteria, excluding keywords referring to specific food sources to ensure broader coverage of the fundamental microbiological literature investigating GL–bacteria interactions rather than food-specific effects. The detailed list of keywords and their combinations for each literature search is presented in Supplemental Table 1. Manual searches based on references of selected reviews were performed to supplement the electronic search 1.

### Eligibility criteria and selection

Original articles and reviews that met the following criteria were included in the present systematic review: (1) human studies, (2) preclinical studies representative of humans (both animal and *in vitro* models), (3) use of GLs or GL components or foods rich in GLs, and (4) study of the gut microbiota composition or bacteria–GL interactions (outcomes). The present review thus combines human and animal studies to provide a complementary view of plant glycolipids–gut microbiota interactions in this emerging research field. All studies included in the present review involve bacterial species that are known to be part of, or associated with, the human gut microbiota. Studies on plant metabolism were excluded. Only literature published in English or French were considered. Two authors (AC, CV) independently screened the titles and abstracts of the articles using the search strategy to identify those that met the inclusion criteria outlined above. The same authors (AC, CV) assessed the full texts of preselected articles for eligibility. Disagreements were resolved by consensus.

### Data extraction

One author (AC) extracted relevant data from the included studies. The extracted information was verified by a second author (CV). The following information was extracted from each included study: model, type and source of GLs, treatment or experimental conditions, and microbiota outcomes (modulation or bacterial degradation). In Table 1, selected studies are described and classified as *in vitro*, animal, and human studies.

**Table 1.** Main characteristics of studies included in the present review.

References	Model	Type and source of glycolipids (GLs)	Treatment/condition	Gut microbiota modulation by GLs	Degradation of GLs by gut microbiota
***In vitro* studies**					
[26]	Gram-positive, Gram-negative bacteria and fungi	Mono-glucosyl and mono-galactosyl diglycerides (synthetic)	*In vitro* antimicrobial assays against Gram+/Gram− bacteria and *Candida* spp.	No antifungal activity; structure-dependent antibacterial activity	NA
[27]	Gram-positive, Gram-negative bacteria and fungi	Monogalactosyl diglycerides (MGDG, synthetic)	*In vitro* antimicrobial assays against Gram+/Gram− bacteria and Candida spp.	Similar to [26] No antifungal activity; antibacterial activity associated with C8:0 acyl chains and 1,2-distribution	NA
[28]	Aerobic and anaerobic strains of bacteria (phylum Firmicutes): *Bacillus aryabhattai, Clostridium symbiosum, Enterococcus gilvus, Eubacterium rectale*	_13_C_6_-isotopically labeled sulfoquinovose (SQ)	Growth of environmental and gut Firmicutes on SQ		Transaldolase pathway for SQ degradation
[29]	Bioinformatic and biochemical analysis of bacterial SQ degradation pathways	SQ	*In vitro* enzymatic characterization of sulfoglycolysis pathways	NA	Sulfo-TK, sulfo-ASMO, and sulfo-EMP2 pathways
[30]	Bacteria isolated from forest leaf mold (*Klebsiella* sp. and *Agrobacterium* sp.)	^13^C-SQ (synthetic)	Growth of specific bacteria on SQ	NA	SQ bacterial degradation with delayed sulfate release
[23]	YihQ enzyme from *E. coli*	SQDG/SQ	*In vitro* characterization of *E. coli* YihQ	NA	Complete enzymatic hydrolysis of spinach SQDG to SQ.
**Animal studies**					
[31]	Interleukin-10-deficient mice (conventional and gnotobiotic) Conventional: -Feeding experiment *n* = 24 -Force-feeding *n* = 34 Gnotobiotic (SIHUMI mice) *n* = 48	SQDG-rich spirulina and SQ (commercial source)	Dietary supplementation (3 weeks) or oral gavage	Reduced *B. wadsworthia* and altered β-diversity in spirulina-fed mice. No cecal microbiota changes with SQ gavage in conventional mice. SQ increased *B. wadsworthia* in orally gavaged gnotobiotic mice, with no change in overall community composition.	Fecal microbiota converted SQ to sulfide
[32]	Male adult Sprague–Dawley rats with diet-induced non-alcoholic fatty liver disease (NAFLD); *n* = 44	Spinach (*Spinacia oleracea*)	Normal or high-fat diet supplemented with spinach (2.5 or 5%) in NAFLD rats during 5 weeks	Increased *Bifidobacteriu*m and *Lactobacillus* in spinach-supplemented groups	NA
[33]	Sprague–Dawley rats; *n* = 16 (8/group)	Thylakoids from fresh baby-spinach leaves	Control diet (chow enriched with rapeseed oil) vs thylakoid diet (chow enriched with thylakoid-oil suspension) during 10 d	Modulation of *Lactobacillus* species in ileal and colonic mucosa; no change in overall diversity (Shannon–Wiener diversity index)	NA
[34]	Male wild-type C57BL/6 mice; *n* = 45 (5/group)	Gut microbiota-associated GLs	Antibiotic treatment (ampicillin, vancomycin, neomycin, metronidazole) in mice for 2 weeks		Reductions of MGDG and DGDG in antibiotic-treated mouse feces (>10-fold) *vs* control.
**Human studies**					
[35]	Simplified human intestinal microbiota (SIHUMI)	SQ (synthetic)	SIHUMI cultures incubated with SQ, with or without *B. wadsworthia*	NA	SQ degraded to DHPS; DHPS further converted to sulfide by *B. wadsworthia*
[36]	Human fecal microcosms and co-cultures	SQ (synthetic)	Anaerobic microcosms containing human vegetarian fecal samples and incubations with SQ or control substrates Co-cultures of *E. rectale* and *B. wadsworthia* with SQ	NA	Cooperative SQ metabolism by *E. rectale* and *B. wadsworthia*, producing acetate and H₂S
[37]	Overweight women (*n* = 34)	Spinach thylakoids	12-week thylakoid or control dietary supplementation (blueberry drink with or without thylakoids)	Increased total bacteria and *B. fragilis* group in the thylakoid group; no major shifts in other taxa	NA

DGDG: digalactosyldiacylglycerol; DHAP: dihydroxyacetone phosphate; DHPS: 2,3-dihydroxy-propane-1-sulfonate; GL: glycolipid; MGDG: monogalactosyldiacylglycerol; MIC: minimum inhibitory concentration; NA: not applicable; OTU: operational taxonomic unit; NMR: nuclear magnetic resonance; SQ: sulfoquinovose; sulfo-ASMO: sulfoglycolytic alkanesulfonate monooxygenase; sulfo-EMP: sulfoglycolytic Embden–Meyerhof–Parnas; sulfo-TK: sulfoglycolytic transketolase; TC: taurocholate.

## Results & discussion

### Literature search

Figure 2 presents the flowchart of selected studies for search 1 and search 2. A total of 345 articles were retrieved from both searches. For search 1, an initial sample of 267 records was identified: 265 from the Web of Science database and 2 from manual searches. Those records were screened for title and abstract based on exclusion criteria (previously mentioned), resulting in the removal of 256 records. Then, 11 records were full-text analyzed; 2 records were excluded, i.e. one article used an inappropriate model (ruminant), and another did not use GLs; 3 reviews were fully assessed and allowed to include 2 original articles from reference lists, after which reviews were excluded. Finally, 8 original articles were selected from search 1 for the systematic review. For search 2, 78 records were identified from the WOS database. One duplicate was subsequently removed, and the remaining 77 records were screened for title and abstract based on exclusion criteria, resulting in the removal of 71 records. Six records were then fully reviewed, and 1 record was excluded because of an inappropriate model (plant metabolism of bacteria). Five articles from search 2 were definitely included in the present review. Finally, 13 articles were selected from both searches for the systematic review (Figure 2), of which six were *in vitro* model studies,^23,26-30^ 4 were animal studies,^31-34^ and 3 were human-based studies.^35-37^

**Figure 2.**
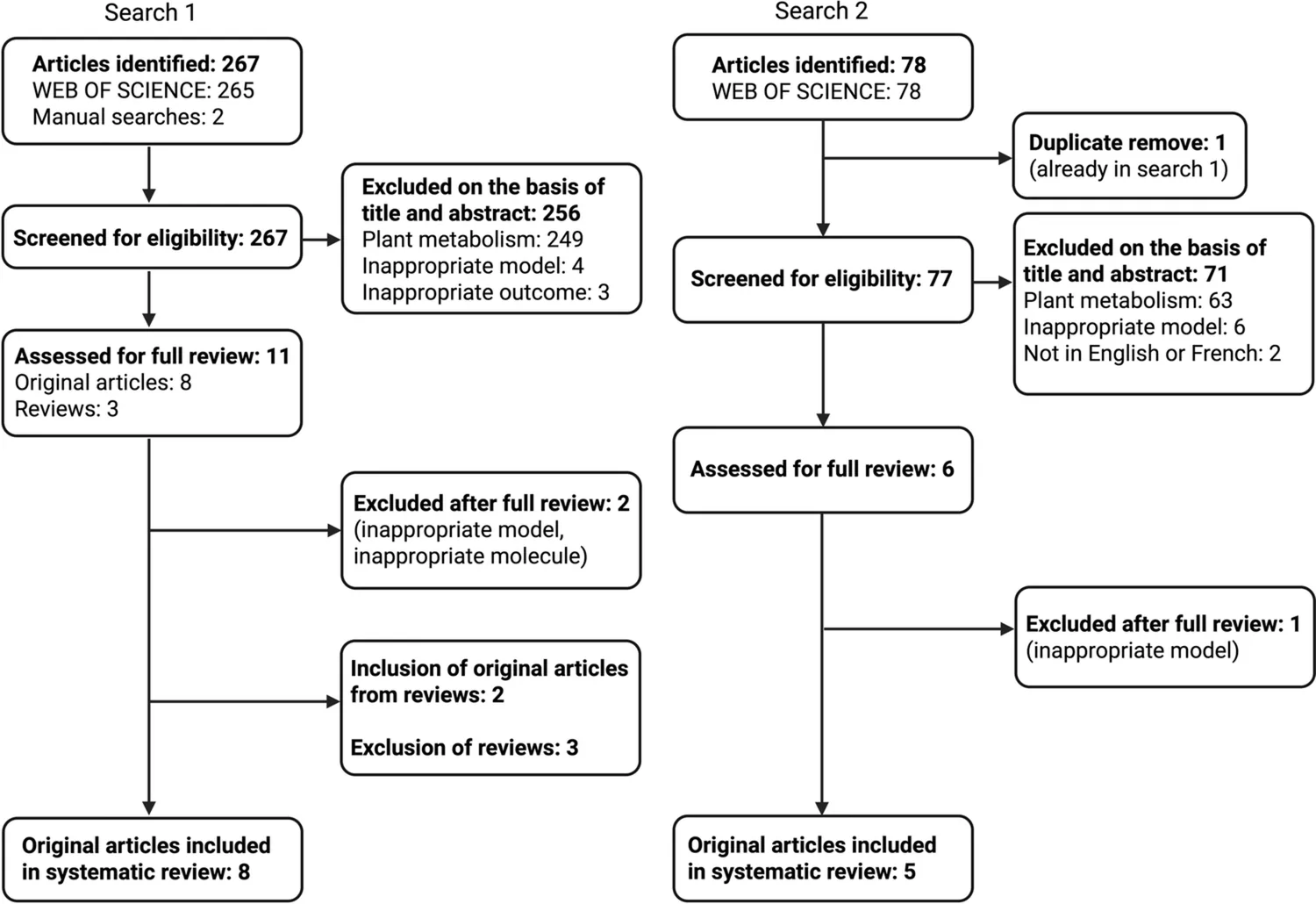
Flowchart of the literature search describing the selection process.

### Studies characteristics

The majority of *in vitro* model studies (*n* = 4) deal with SQDG or SQ and their degradation pathways by selected and isolated intestinal bacteria^23^ ^,^ ^28-30^ and the others (*n* = 2) focused on the antibacterial and antifungal activities of GLs^26^ ^,^ ^27^ (Table 1). Animal studies were performed using rodent models, either Sprague–Dawley rats (*n* = 2)^32^ ^,^ ^33^ or mice (wild type or innate immune-deficient; *n* = 2,^31^ ^,^ ^34^ Table 1). Regarding the human-based studies, one randomized controlled trial (RCT) conducted on overweight women with thylakoid supplementation analyzed the fecal microbiota^37^, and the other two rested on cultures of the human microbiota (simplified human intestinal microbiota -SIHUMI- or co-cultures)^35^ ^,^ ^36^ (Table 1).

### GL supplementation using spinach or spirulina extract modulates gut microbiota composition and function

Several preclinical studies in rodents investigated the effects of GL either from spinach thylakoids, spinach or SQDG-rich spirulina on the gut bacterial composition (Figure 3). One study focused on the microbiota composition of different parts of the gut after 10 d of spinach thylakoid supplementation in Sprague–Dawley rats.^33^ Rats were fed either chow enriched with a thylakoid-oil suspension or chow enriched with rapeseed oil (control diet). 16S rDNA sequence analysis showed significant increase in *Lactobacillus* in the ileal mucosa in the thylakoid group compared with the control group, which was associated with a higher incidence of *Lactobacillus reuteri* and a lower incidence of *Lactobacillus johnsonii* in the ileal mucosa. *L. johnsonii* was also significantly reduced in the colonic mucosa in the thylakoid group compared with the control group.

**Figure 3.**
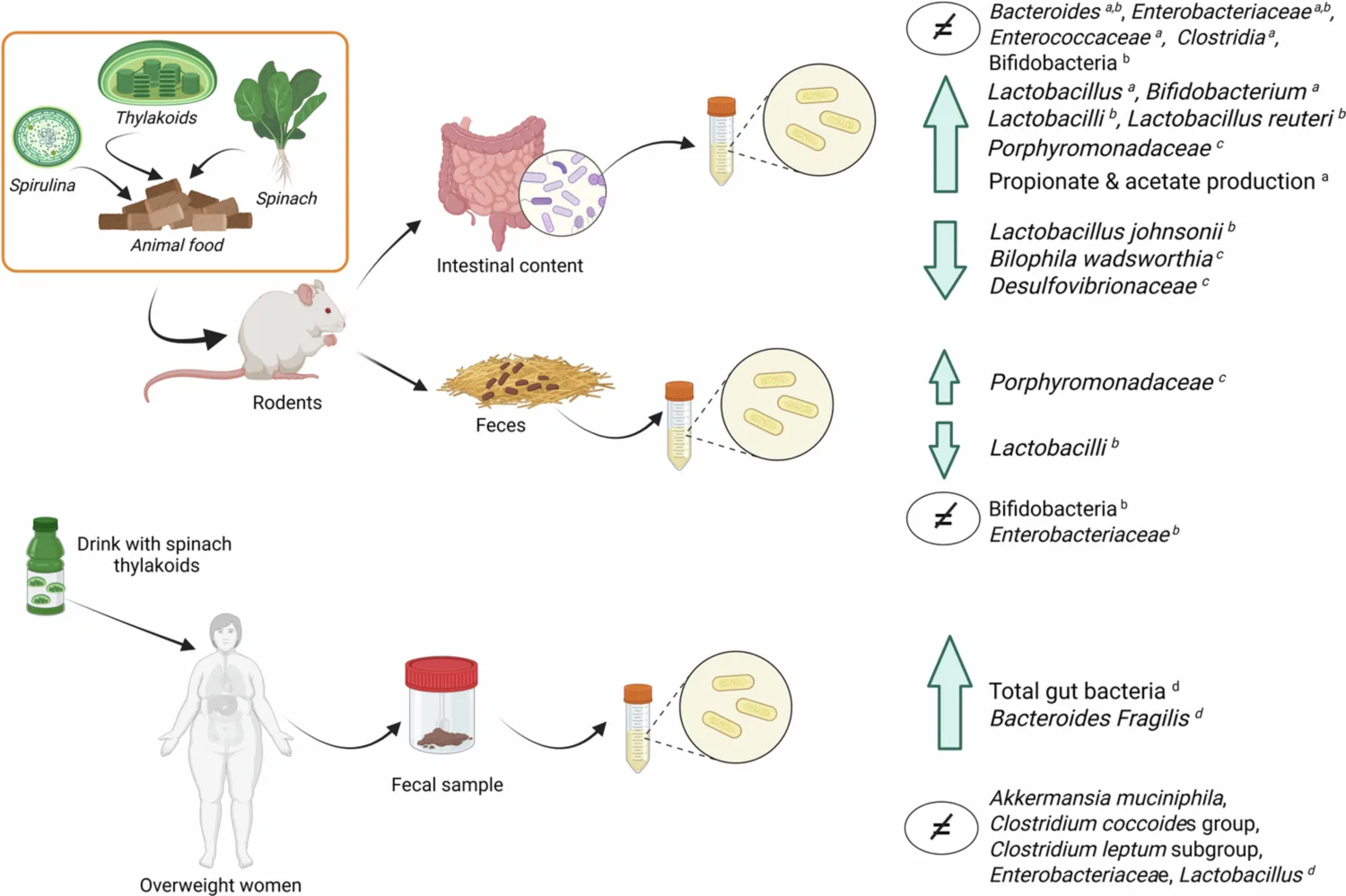
Effects of dietary supplementation with GL-rich plant-based foods on the gut microbiota composition in existing rodent and clinical studies. (a) from (Elvira-Torales *et al.* ^32^); (b) from (Montelius *et al.* ^33^); (c) from (Burkhardt *et al.* ^31^); (d) from (Stenblom *et al.* ^37^). Created with BioRender.com.

*Lactobacilli* are Gram-positive, facultative anaerobic bacteria of the phylum “Firmicutes” which metabolize carbohydrates to produce lactic acid.^38^ *L. reuteri* is known to produce antimicrobial molecules (organic acids, ethanol), which (1) inhibit the colonization of pathogenic microbes and remodel the commensal microbiota composition in the host and (2) have inhibitory effects on abdominal fat accumulation and age-related weight gain in mice irrespective of their baseline diet.^39^ ^,^ ^40^ *L. johnsonii* strain is known to be a beneficial bacterium in the human intestine, particularly its peptidoglycan can inhibit the pro-inflammatory IL-12 production,^41^ contributing to the maintenance of host intestinal homeostasis. Therefore, the findings of Montelius *et al*. suggest that thylakoid supplementation may induce selective shifts within *Lactobacillus* populations rather than a uniform increase, potentially leading to changes in microbiota functionality. While the increase in *L. reuteri* could be associated with antimicrobial and metabolic effects, the decrease in *L. johnsonii*, known for its anti-inflammatory properties, indicates a complex and strain-dependent response. This highlights the importance of considering both taxonomic and functional aspects when interpreting microbiota modulation.

Another study conducted in male adult Sprague–Dawley rats with high-fat diet-induced non-alcoholic fatty liver disease (NAFLD) also showed an increase of up to 9% in *Lactobacillus* counts in rats fed a high-fat diet supplemented with spinach compared to rats fed the non-supplemented high-fat diet.^32^ A slight increase in *Bifidobacterium* counts (2%) was also observed in rats fed either the spinach supplemented normal or high-fat diet. Moreover, the consumption of spinach-supplemented diets (normal or high-fat) resulted in increased propionate production, and an increase in acetate production by 44% was observed after the consumption of the high-fat diet enriched with spinach. Short-chain fatty acids (SCFAs), mainly acetate (C2), propionate (C3), and butyrate (C4), are produced by the gut microbiota during the fermentation of partially and nondigestible polysaccharides and are largely described to improve gut health and functionality through a number of local effects, ranging from the maintenance of intestinal barrier integrity, mucus production, and protection against inflammation.^42^ Thus, the consumption of spinach (naturally rich in GLs) was associated with an improved gut microbiota functionality through an increase in the production of some SCFAs. This observation is consistent with changes observed in the microbiota composition, as *Lactobacillus* and *Bifidobacterium* spp. have been previously reported as SCFA-producing bacteria.^43^ Furthermore, other groups of bacteria can display detrimental effects, especially *Enterobacteriaceae* , which contain specific molecular components called microbe-associated molecular patterns (MAMPs) that interact with the receptors on immune cells to trigger inflammation.^42^ The two above-mentioned studies in rats reported no impact of supplementation of spinach on *Enterobacteriaceae* abundance,^32^ ^,^ ^33^ suggesting that spinach supplementation rich in GLs may not promote the expansion of potentially pro-inflammatory bacterial groups.

Burkhardt *et al*. investigated the effects of another source of GLs, i.e. spirulina which is particularly rich in the SQDG. A 21-d spirulina supplementation in conventional IL-10^−^^/−^ mice significantly decreased the cecal cell number of *Bilophila wadsworthia* and the relative abundance of members of *Desulfovibrionaceae* compared to the control group^31^ (Figure 3). *B. wadsworthia* is known to promote higher inflammation and intestinal barrier dysfunction,^44^ and bacteria of the *Desulfovibrionaceae* family are opportunistic pathobionts.^45^ Such decreases in these pathobionts are thus considered beneficial effects of spirulina. In parallel, IL-10^−/−^ mice were daily gavaged with SQ during 3 weeks, and no change in cecal abundance of *B. wadsworthia* nor α- and β-diversity was observed after SQ gavage compared to the vehicle control. Thus, SQ seemed to play no role in the effects on microbiota composition observed with spirulina supplementation. However, SQ, but not SQDG, was tested in the forced-fed study, therefore a specific effect of the complete SQDG molecule as present in green photosynthetic organisms cannot be ruled out and still deserves to be explored. Moreover, these results were obtained in the cecum, which does not include colonic bacteria and thus excludes the potential impact of colon metabolism and the microbiota. Interestingly, β-diversity analysis of the fecal microbiota showed differences in the microbiota composition between spirulina-fed and control mice, with higher abundance of *Porphyromonadaceae* after 18 d of spirulina consumption. In the NU-AGE study, an elevated abundance of fecal *Porphyromonadaceae* was recently associated with a reduced visceral fat mass and healthier metabolic profile in elderly.^46^ Therefore, further studies should investigate the role of GL metabolism in cardiometabolic health and diseases.

In humans, overweight women who daily consumed a blueberry drink supplemented with spinach thylakoids (5 g Appethyl®) during 12 weeks showed significant increased fecal amounts of total gut bacteria and *Bacteroides fragilis* compared to the control group^37^ (Figure 3). *B. fragilis* constitutes less than 1% of the colonic microflora but it produces a specific capsular polysaccharide that is sufficient to correct some of the immune system deficiencies and to prevent intestinal inflammation in animal models of colitis.^47^ No significant differences were found in either thylakoid or control group regarding number of 16S rRNA gene copies of *Akkermansia muciniphilia*-like bacteria, *Clostridium coccoides* group, *Clostridium leptum* subgroup, *Enterobacteriaceae* or *Lactobacillus* at endpoint compared to baseline. Discrepancies with previous rat studies showing notably increased *Lactobacillus* after spinach supplementation may be explained by differences in microbiota responses between human *vs* rodents, or by differences in the diet (more diversified in humans) or by interactions between blueberry bioactives and spinach thylakoids in the present supplementation. However, such differences and the spinach thylakoid-driven modification of *B. fragilis* observed in women are not discussed by the authors. The quality assessment of the risk of bias of this RCT was conducted independently by two authors (MCM, CV) using the Cochrane tool^48^ and considered it “low to moderate risk” because 3 items (random sequence generation; blinding of participants; other bias) were judged as “low risk” and 4 items (allocation concealment; blinding of outcome assessment; incomplete outcome; selective reporting) were judged as “unclear”. Further well-designed and adequately powered human studies are needed to confirm these findings and to better characterize the impact of dietary interventions based on GL-rich food sources on gut microbiota composition and function.

### MGDG as an antimicrobial agent

Cateni *et al.* have tested the antibacterial activity against *Staphylococcus aureus*, *Enterococcus faecalis*, *Bacillus subtilis*, *Escherichia coli*, *Pseudomonas aeruginosa*, *Mycobacterium tuberculosis*, and the antifungal activity against *Candida albicans* and *Candida pseudotropicalis* of several monoglucosyl and monogalactosyl diglycerides according to different lengths of FA acyl chains (from C8 to C18).^26^ ^,^ ^27^ In both studies, no inhibitory activity was exhibited against *Candida* spp by any type of MGDG. The authors showed that the antibacterial activity of MGDG is influenced by several factors: the chain length of the FA, saturation/unsaturation of the FA, 1,2-distribution of the FA, and nature of the carbohydrate residue. Briefly, the maximum antibacterial activity is observed with the octanoyl chain (C8:0) but also when compounds contain the C8:0 at sn-1 and unsaturated FAs at the sn-2 position of glycerol. No antibacterial activity was observed with saturated (>C12) and monounsaturated FAs, whereas compounds characterized by the presence of two linoleic acids (C18:2, PUFAs) in the positions sn-1 and sn-2 of glycerol displayed inhibitory activity against some bacterial strains. Glucosyl diglycerides present more potent antibacterial effect compared to galactosyl diglycerides. However, a compound with a galactosyl residue bearing three unsaturations (C18:3) exhibited activity against *S. aureus*, whereas the analog compound with a glucosyl residue did not show any antibacterial activity. Therefore, MGDG has significant antibacterial effects but does not present antifungal properties. It remains to be elucidated if MGDG can be present along the intestinal tract and in which amount to display such effects on gut bacteria.

Importantly, a recent study reported a deep analysis of gut-associated lipids in male wild-type C57BL/6 mice.^34^ Analytical approaches using LC‒MS/MS and 16S rRNA sequence analyses were performed on fecal samples from mice treated with antibiotic-cocktails (Abx) for two weeks versus the control (no Abx). Among a long list of identified lipids, MGDG and DGDG were detected and quantified in both groups. Additionally, the abundances of MGDG and DGDG were decreased by more than 10-fold in the group of Abx treated mice compared to the control. Such results reveal that GLs can be present in the colon of mice and that GL abundance is impacted by gut bacteria. Authors explain results of MGDG by stipulating that MGDG are distributed in anaerobic bacteria, including intestinal *Clostridium* species. Nevertheless, the authors did not describe the type of food or food intake of the animals, and the presence of dietary GLs in both animal food and feces cannot be excluded.

### Degradation of SQDG and derived SQ by gut bacteria

SQDG is a ubiquitous sulfolipid present in the photosynthetic membranes of plants, algae, and cyanobacteria.^29^ SQDG represents one of the most abundant organic sulfur compounds in the biosphere and can represent greater than 25% of the total lipids in common dietary leafy green vegetables such as spinach, lettuce, and green onion.^36^ SQ is the polar headgroup of the SQDG and contains a chemically inert sulfonate C−S bond unlike other carbohydrates.

Bacterial degradation of SQ is named sulfoglycolysis, for which several pathways have been reported to date.^23^ ^,^ ^28^ ^,^ ^29^ Figure 4 illustrates the different glycolytic pathways, including involved bacteria and the sulfur-containing by-products. Among the first that were discovered, the sulfo-EMP (Embden–Meyerhof–Parnas) and sulfo-ED (Entner–Doudoroff) pathways occur in Gram-negative bacteria, and recently the sulfo-TAL pathway (also called Transaldolase pathway) was described in diverse Gram-positive bacteria.^28^ ^,^ ^29^ Indeed, the latter was identified in phylum Firmicutes bacteria, and occurs especially in human gut *Enterococcus*, *Clostridium*, and *Eubacterium* strains.^28^ Liu *et al.* discovered very recently additional sulfoglycolysis pathways: (1) the sulfo-EMP2 pathway, a variant of the sulfo-EMP pathway in Gram-positive bacteria, (2) the sulfo-TK (Transketolase) pathway only in strict anaerobic fermenting bacteria (including intestinal Firmicutes), and (3) the sulfo-ASMO (alkanesulfonate monooxygenase) pathway only in aerobic soil α-proteobacteria.^29^ In the intestinal tract, the sulfo-EMP, sulfo-ED, and sulfo-TAL pathways allow the incorporation of three out of six SQ carbons into cellular metabolism, whereas the sulfo-TK pathway allows the incorporation of four SQ carbons.^29^ Indeed, bacterial SQ degradation through all glycolytic pathways generates diverse by-products that fuel carbon and energy metabolism of bacteria. The sulfo-EMP2, sulfo-ED, and sulfo-TAL pathways produce C3 sulfonate by-products (2,3-dihydroxypropane-1-sulfonate, DHPS or sulfolactate, SL), while the sulfo-TK pathway produces C2 sulfonate by-products (isethionate or sulfoacetate)^23^ ^,^ ^28-30^ (Figure 4). The sulfonate products of the sulfo-TK pathway are intermediates in the pentose phosphate pathway, which are substrates for both respiration and fermentation.^29^ The C3 sulfonates can be catabolized into hydrogen sulfide (H_2_S), which is an important intestinal metabolite that modulates the activity of mitochondria in host epithelial cells,^49^ but also of microbial cells.^50^ However, at high concentrations, H_2_S can chemically deteriorate the mucosal barrier of the gastrointestinal tract and decrease ATP production through the inhibition of cytochrome C, potentially leading to cell death.^51^ Thus, the complete degradation of SQ through these pathways requires the involvement of other bacteria capable of sulfonate C‒S cleavage.

**Figure 4.**
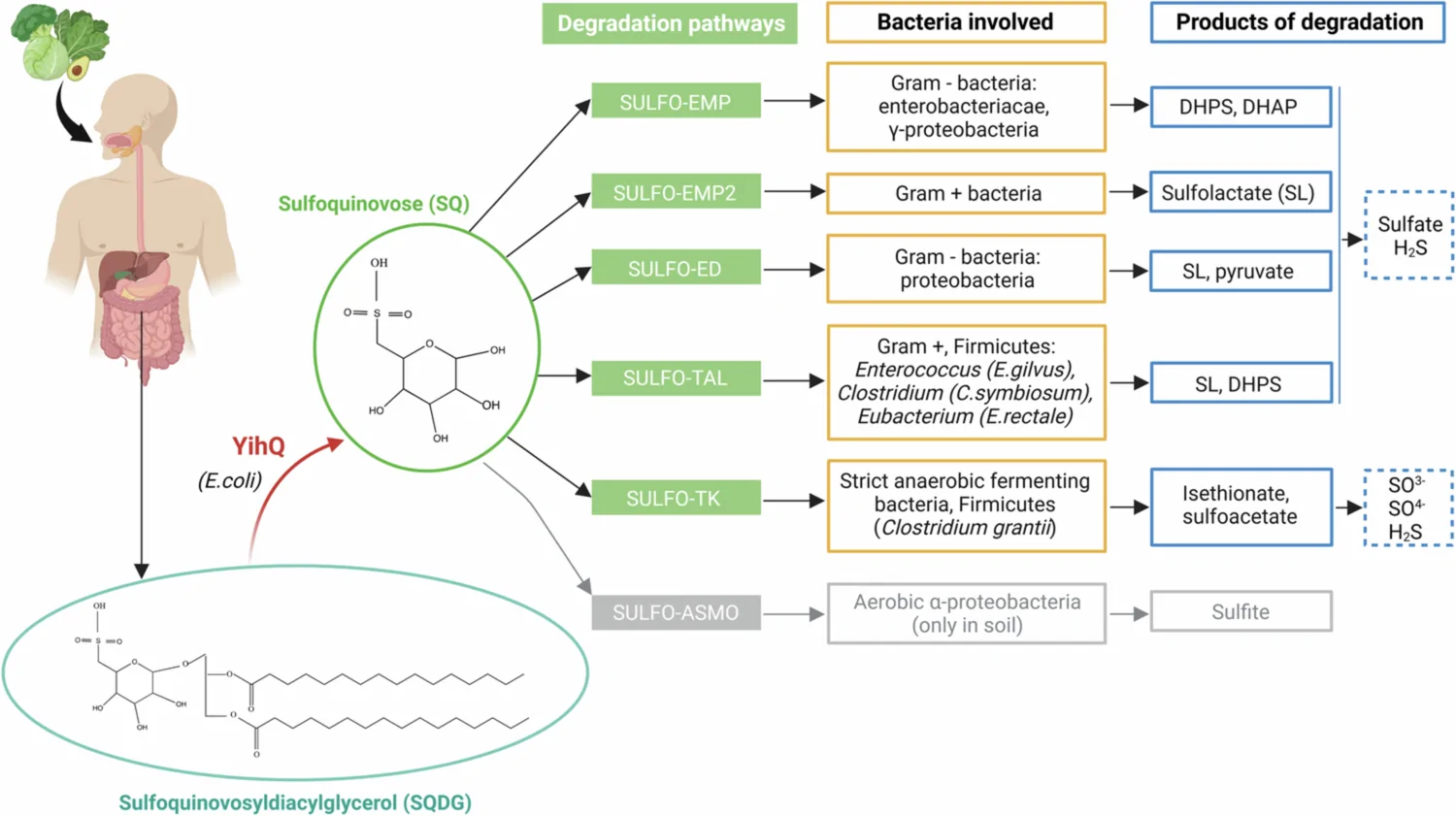
Schematic bacterial degradation pathways of sulfoquinovosyldiacylglycerol (SQDG) and sulfoquinovose (SQ). ASMO: alkanesulfonate monooxygenase; DHAP: dihydroxyacetone phosphate; DHPS: dihydroxypropane sulfonate; ED: Entner–Doudoroff; EMP: Embden–Meyerhof–Parnas; H_2_S: hydrogen sulfide; SL: sulfolactate; SO^3^^−^: sulfur trioxide; SO^4^^−^: sulfate; TAL: transaldolase; TK: transketolase; YihQ: sulfoquinovosidase. Created with BioRender.com.

Recent studies using the human microbiota nicely illustrated the combined bacterial action required for total SQ degradation. Haange *et al*. incubated SQ in anaerobic cultures of simplified human intestinal microbiota (SIHUMI) with or without *B. wadsworthia.* ^35^ They observed a significant decrease in SQ in SIHUMI+SQ cultures (48 h), concomitantly with an increase in DHPS (without sulfide production). The quantification of sulfide was only possible when *B. wadsworthia* was present in the SIHUMI+SQ cultures (24–48 h), showing that bacteria from SIHUMI can degrade SQ into DHPS, which is further catabolized by *B. wadsworthia* into H_2_S. Furthermore, Hanson *et al.* constructed microcosms with fecal samples from vegetarian individuals, which were incubated with SQ or control substrates.^36^ SQ was completely consumed within 20 h concomitant with a transient accumulation of DHPS between 20 h and 52 h. An increased production of H_2_S was also observed, with a peak at 96 h, with a gradually increased of acetate over 144 h compared to control microcosms. The degradation of both SQ and DHPS was accompanied by specific changes in bacterial composition, as revealed by 16S rRNA analysis. Under these conditions, the abundances of *Eubacterium rectale* and *Bilophila wadsworthia* were the most strongly increased. To confirm that SQ was cooperatively metabolized in the microcosms, mainly by *E. rectale* and *B. wadsworthia*, the authors co-cultured isolates of *E. rectale* and *B. wadsworthia* with SQ and showed that *E. rectale* grew by fermenting SQ and produced DHPS, which was rapidly consumed after co-inoculation of *B. wadsworthia* leading to the production of H_2_S. Using metatranscriptomic and proteomic approaches on fecal microcosms and culture experiments, they demonstrated that *E. rectale* ferments SQ in the gut *via* the sulfo-TAL pathway to produce DHPS, which is then desulfonated by *B. wadsworthia* to H_2_S. Altogether, these findings highlight the diversity of mechanisms through which gut bacteria degrade and use SQ compounds as nutrient sources.

While SQDG is the primary source of SQ that supplies the different sulfoglycolytic pathways, the mechanism by which SQ is cleaved from SQDG remains an important issue. Recently, Speciale *et al* *.* were the first to identify and characterize a bacterial sulfoquinovosidase.^23^ This enzyme, named YihQ and identified in *E. coli,* was incubated with SQDG isolated from spinach, and the LC/MS analysis of the reaction mixture revealed complete conversion to SQ. Therefore, YihQ is involved in the degradation of sulfoquinovosides, i.e. hydrolysis of SQDG to SQ, and is considered as the first gateway enzyme to sulfoglycolytic pathways. These findings highlight the key role of bacterial sulfoquinovosidases in enabling the utilization of dietary SQDG, thereby contributing to sulfoglycolytic metabolism within the gut microbiota.

### Strengths and limitations

To the best of our knowledge, this is the first review summarizing and analyzing the existing literature on dietary plant GLs and the gut microbiota. The present review highlights the significant impact of GLs from plant thylakoids or GL-rich green vegetables on the composition of gut bacteria and describes the bacterial mechanisms of GL degradation. The present review was conducted following the Cochrane guidance to ensure a robust and systematic methodology. The results from only three human studies, including one RCT with a low to moderate risk of bias, were included in the present review, showing the lack of evidence from human trials on this topic. Despite some discrepancies between studies in microbiota modifications due to the different models and sources of GL, several GL-induced changes observed in gut microbiota composition were consistent. Additionally, most studies have explored the effects of GL-rich extracts (spinach, spirulina), but such extracts or matrices are known to contain other bioactive molecules (including polyphenols and chlorophyll) whose own effects and synergistic effects cannot be ruled out. More dedicated studies with mechanistic research and clinical evidence are required for future work on this topical issue.

## Conclusion

Dietary supplementations with green plant and microorganism extracts rich in GLs are reported to have a positive impact on the gut microbiota composition by promoting specific species, such as *L. reuteri* and *Bifidobacterium* in rodents and *Bacteroides* in humans, which are known to have beneficial effects on host health and microbiota eubiosis but also by reducing potential pathobionts. Supplementation with GL-rich spinach is associated with the production of some SCFAs known as primary energy sources for colonic epithelial cells and for their role in the host mucosal immune response. Moreover, MGDG showed significant antibacterial properties, especially against pathogen bacteria. Growing literature provides detailed pathways for bacterial degradation of SQDG and derived SQ. Therefore, plant GLs could be tough prebiotic candidates. Further studies should explore GL-microbiota crosstalk and its metabolic and clinical implications for the host more deeply.

## Supplementary Material

Supplementary Material Supplemental Table 1.docx

## Data Availability

Data will be made available on request.
