# Supplementary material for: Exploring the interplay between dietary plant glycolipids and gut microbiota: A systematic review
Source: Gut Microbes Rep. 2026 Sep 26;3(1):2676398. doi: 10.1080/29933935.2026.2676398 (PMC13618843; doi:10.1080/29933935.2026.2676398)
Supplement: Supplementary file 1 [file KGMR_A_2676398_SM1045.docx]

**Supplemental Table 1.** Search strategy.

| Database | Web Of Science  Search 1 | Web Of Science  Search 2 |
| --- | --- | --- |
| Search Period | to March 20, 2023 | to March 20, 2023 |
| Search Terms | 1. avocado 2. cucumber 3. bean 4. spinach 5. leek 6. broccoli 7. asparagus 8. pea 9. lettuce 10. pistachio 11. “pea vine” 12. parsley 13. coriander 14. mint 15. cabbage 16. “green vegetables” 17. “green lea*” 18. PUFA 19. polyunsaturated 20. "omega-3" 21. “omega 3” 22. “n-3” 23. ALA 24. linolen* 25. thylakoid* 26. chloroplast* 27. galactolipid* 28. monogalactosyldiacylglycerol* 29. monogalactosyl-diacylglycerol* 30. “monogalactosyl diglyceride*” 31. MGDG 32. digalactosyldiacylglycerol* 33. digalactosyl-diacylglycerol* 34. “digalactosyl diacylglycerol*” 35. “digalactosyl diglyceride*” 36. DGDG 37. sulfoquinovosyldiacylglycerol* 38. sulfoquinovosyl-diacylglycerol* 39. “sulfoquinovosyl diacylglycerol*” 40. “sulfoquinovosyl diacylglyceride*” 41. SQDG 42. sulfoquinovose 43. “gut microbio*” 44. “intestinal microbio*” 45. microbiota 46. bacteria 47. 1 or 2 or 3 or 4 or 5 or 6 or 7 or 8 or 9 or 10 or 11 or 12 or 13 or 14 or 15 or 16 or 17 or 18 or 19 or 20 or 21 or 22 or 23 or 24 48. 25 or 26 or 27 or 28 or 29 or 30 or 31 or 32 or 33 or 34 or 35 or 36 or 37 or 38 or 39 or 40 or 41 or 42 49. 43 or 44 or 45 or 46 50. 47 and 48 and 49 | 1. thylakoid* 2. chloroplast* 3. galactolipid* 4. monogalactosyldiacylglycerol* 5. monogalactosyl-diacylglycerol* 6. “monogalactosyl diglyceride*” 7. MGDG 8. digalactosyldiacylglycerol* 9. digalactosyl-diacylglycerol* 10. “digalactosyl diacylglycerol*” 11. “digalactosyl diglyceride*” 12. DGDG 13. sulfoquinovosyldiacylglycerol* 14. sulfoquinovosyl-diacylglycerol* 15. “sulfoquinovosyl diacylglycerol*” 16. “sulfoquinovosyl diacylglyceride*” 17. SQDG 18. sulfoquinovose 19. “gut microbio*” 20. “intestinal microbio*” 21. microbiota 22. bacteria 23. avocado 24. cucumber 25. bean 26. spinach 27. leek 28. broccoli 29. asparagus 30. pea 31. lettuce 32. pistachio 33. “pea vine” 34. parsley 35. coriander 36. mint 37. cabbage 38. “green vegetables” 39. “green lea*” 40. PUFA 41. polyunsaturated 42. “omega-3” 43. “omega 3” 44. “n-3” 45. ALA 46. linolen* 47. 1 or 2 or 3 or 4 or 5 or 6 or 7 or 8 or 9 or 10 or 11 or 12 or 13 or 14 or 15 or 16 or 17 or 18 48. 19 or 20 or 21 or 22 49. 23 or 24 or 25 or 26 or 27 or 28 or 29 or 30 or 31 or 32 or 33 or 34 or 35 or 36 or 37 or 38 or 39 or 40 or 41 or 42 or 43 or 44 or 45 or 46 50. 47 and 48 not 49 |
